# The Influence of UV Light on Photodegradation of Acetylsalicylic Acid

**DOI:** 10.3390/ijms22084046

**Published:** 2021-04-14

**Authors:** Monica Daescu, Miruna Iota, Constantin Serbschi, Alina C. Ion, Mihaela Baibarac

**Affiliations:** 1 Laboratory of Optical Processes in Nanostructured Materials, National Institute of Materials Physics, Atomistilor Street 405A, POB MG 7, 077125 Bucharest, Romania; monica.daescu@infim.ro (M.D.); miruna.iota@infim.ro (M.I.); 2 Faculty of Applied Chemistry & Material Science, University Politehnica of Bucharest, Gh. Polizu Street 1-7, 011061 Bucharest, Romania; ac_ion@yahoo.com; 3 Bioelectronic SRL, Cercelus St 54, 100028 Ploiesti, Romania; bioelectronic90@yahoo.com

**Keywords:** acetylsalicylic acid, photoluminescence, Raman scattering, IR spectroscopy

## Abstract

Photodegradation of the aqueous solutions of acetylsalicylic acid, in the absence (ASA) and the presence of excipients (ASE), is demonstrated by the photoluminescence (PL). A shift of the PL bands from 342 and 338 nm to 358 and 361–397 nm for ASA and ASE in solid state and as aqueous solutions was reported. By exposure of the solution of ASA 0.3 M to UV light, a decrease in the PL band intensity was highlighted. This behavior was revealed for ASA in the presence of phosphate buffer (PB) having the pH equal to 6.4, 7, and 8 or by the interaction with NaOH 0.3 M. A different behavior was reported in the case of ASE. In the presence of PB, an increase in the intensity of the PL band of ASE simultaneously with a change of the ratio between the intensities of the bands at 361–364 and 394–397 nm was highlighted. The differences between PL spectra of ASA and ASE have their origin in the presence of salicylic acid (SAL). The interaction of ASE with NaOH induces a shift of the PL band at 405–407 nm. Arguments for the reaction of ASA with NaOH are shown by Raman scattering and FTIR spectroscopy.

## 1. Introduction

Pharmaceutical compounds have been designed to have a specific mode of action, targeting specific organs, metabolic pathways, or receptors to modulate physiological functions, to treat a disease, and to restore the health of the organism [1]. Non-steroidal anti-inflammatory drugs are a class of drugs that are grouped into drugs with analgesic and antipyretic effects, but also into drugs with anti-inflammatory effects, when used in higher doses [2]. Acetylsalicylic acid (ASA), marketed since 1899 as Aspirin, is known for its anti-inflammatory, analgesic, antipyretic, and antithrombotic effects. Due to these therapeutic effects, its ability to alleviate several symptoms related to common viral infections has been recognized, thus being part of the category of drugs worth testing in patients with COVID-19 [3]. The therapeutic schema involving the use of aspirin has been reported to induce a decrease of the mortality rate for COVID-19 positive patients [4]. However, ASA was eliminated in the case of children and people at risk of bleeding, such as pregnant women or patients with high cardiovascular risk [3]. The mechanism as anti-thrombotic agent of ASA in the SARS-COV-2 infections was reported by Shih-Chang Tsai et al. [5]. The optimal dose of ASA in the therapeutic scheme for the SARS-COV-2 infections was reported to be of 1500 mg/day [6,7,8]. Taking into account that in many infectious diseases, including SARS-COV-2 infections, patients present with temperatures above 36 °C, the influence of temperature on the optical properties of ASA are reported in this work. The reactive nature of the acetyl group found in the structure of aspirin suggests that it may also have off-target chemical reactions that may have a biological effect. As ASA enters a cell in the body, much of it will be transformed due to the hydrolysis reaction with the aqueous solution, leading to the formation of salicylic acid (SAL). This reaction, but also others, represents a possibility to use high concentrations of SAL in the cell, which interact with cell molecules and thus disrupt their activity [9]. In this context, we note that the pharmaceutical product containing acetylsalicylic acid and excipients (ASE) is administered for systemic sclerosis [10], ovarian cancer [11], dysphagia [12], prevention of myocardial infarction [13], atrial fibrillation [14], hepatocellular carcinoma [15], Candida parapsilosis [16], venous thromboembolism [17], and for its anti-tumor effect [18]. 

ASA is a compound containing carboxylic and ester functional groups, the latter being highly vulnerable to hydrolysis under various hydrolytic conditions [19]. Stability testing of pharmaceutical products represents an important part in the drug development process because it provides evidence on how the quality of a drug substance varies with time, under the influence of a variety of environmental factors, such as temperature, humidity, and light [20]. Over the decades, many characterization techniques have been used for the detection of ASA, e.g., UV-VIS absorption spectroscopy [21,22], fluorescence [21,23], powder X-ray diffraction (PXRD) [24,25], FTIR spectroscopy [26], FT-Raman spectroscopy [27], X-ray photoelectron spectroscopy (XPS) [28], nuclear magnetic resonance (NMR) spectroscopy [29], high-performance liquid chromatography (HPLC) [30,31], and cyclic voltammetry (CV) [32]. 

According to reference [33], the degradation process of ASA was studied using liquid chromatography/mass spectrometry (LC/MS), FTIR, and UV spectrophotometric analysis. Another method that highlights the degradation of ASA is electrochemical oxidation, which was validated by ion chromatograph (IC) and gas chromatography-mass spectrometry (GC-MS) [34]. In these cases, the intermediate compounds formed by the process of photocatalytic oxidation or by other mechanisms of ASA degradation, such as hydrolysis, electrophilic addition, electrons transfer, decarboxylation reaction, aromatic ring opening, and radical reaction, have been shown to be more toxic than ASA. In situ monitoring of photodegradation reactions of various drugs has recently been shown to be possible by various optical methods such as UV-VIS absorption spectroscopy and photoluminescence (PL). Arguments for the formation of reaction compounds were often reported by Raman scattering and IR spectroscopy [35,36,37,38]. In order to exemplify this progress, the following issues were reported in previous studies by our team [35,36,37,38]: (1) the influence of oxygen from air on the photodegradation process of azathioprine in the powder and tablet state, respectively [38]; (2) the influence of the phosphate buffer (PB) solution with pH equal to 5.4 on photodegradation of folic acid [37]; and (3) the photodegradation induced by the hydrolysis reaction of melatonin [36] and acetaminophen [35]. In all these studies, PL was found to be a faster optical method in highlighting the photodegradation processes of the above drugs in comparison with the UV-VIS spectroscopy [35,36]. 

For the particular case of ASA, which is marketed as effervescent tablets, known under the commercial name of Bayer Aspirin and Aspirin plus C or as Aspirin hot drink, the knowledge of the behavior of this active compound in aqueous solutions is of interest. Often, the water has a weakly acidic or basic character. Therefore, in this work, new optical evidence concerning the ASA photodegradation in the presence of the PB solutions with the pH ranging between 6.4 and 8 will be shown. Preliminary results concerning the dependence of PL spectra with the temperature of the aqueous solutions of ASA or ASE will be also reported. Using PL, the ASA photodegradation reaction in the presence of alkaline media will be analyzed, too. The understanding of the chemical mechanism that takes place by the interaction of ASA with alkaline medium will be explained, taking into account the variations induced to the vibrational proprieties of ASA that will be presented by Raman scattering and FTIR spectroscopy.

## 2. Results and Discussion

**Figure 1:**
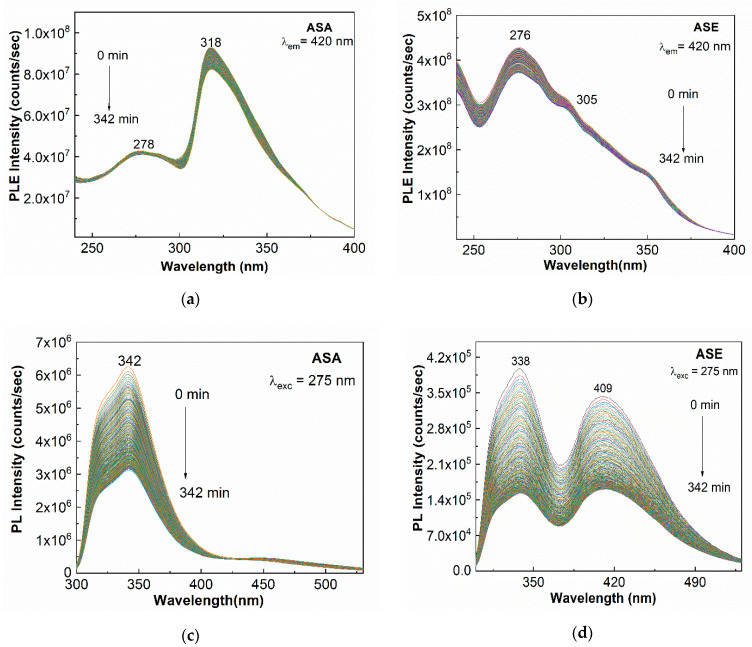
Photoluminescence excitation (PLE) spectra of ASA (**a**) and ASE (**b**), in powder state, recorded at the emission wavelength equal to 420 nm. Photoluminescence (PL) spectra of acetylsalicylic acid (ASA) (**c**) and pharmaceutical product containing acetylsalicylic acid in the presence of excipients (ASE) (**d**), in powder state, recorded at the excitation wavelength equal to 275 nm.

Figure 1 shows the photoluminescence excitation (PLE) and PL spectra of ASA and ASE in powder state.

The PLE spectrum of ASA is characterized by an intense band with maximum at 318 nm and another one of low intensity having maximum at 278 nm (Figure 1a). The ratio between the intensities of the two PLE bands (I_318_/I_278_) is equal to ~2.17. The PLE spectrum of ASE shows an intense band with the maximum at 276 nm, having a shoulder at 305 nm. The ratio between the intensities of the two bands (I_305_/I_276_) is equal to ~0.72 (Figure 1b). The exposure of the two samples, i.e., ASA and ASE in powder state, at the UV light, time of 342 min, induced in PLE spectra a change of the I_318_/I_278_ and I_305_/I_276_ ratios at ~1.95 and ~0.78, respectively. More important variations were observed in the case of PL spectra of ASA and ASE (Figure 1c,d). Before exposure to UV light, PL spectra were characterized in the case of: (1) ASA by an emission band with a maximum of approx. 342 nm and (2) ASE through two emission bands with maxima at approx. 338 and 409 nm. These results were in good agreement with those previously published on ASA and salicylic acid (SAL) when PL bands with maxima at 341 nm and 404 nm, respectively, were reported [21]. The presence of SAL in ASE is a consequence of the partial conversion of ASE into SAL in the presence of water vapors from air adsorbed onto the ASE tablets surface [21]. According to Figure 1c,d, exposure to UV light of the two samples for 342 min induced, in the case of (1) ASA, a gradual decrease of the PL band intensity with the maximum at 342 nm from 6.25 × 10^6^ counts/sec to 3.15 × 10^6^ counts/sec and (2) ASE, a progressive decrease in the intensity of the PL bands at 338 and 409 nm from 3.9 × 10^5^ and 3.4 × 10^5^ counts/sec to 1.5 × 10^5^ and 1.6 × 10^5^ counts/sec, respectively. The choice of exposure time to UV light took into account the instability of ASA in different biological fluids, for which it was known that the ASA half-life by hydrolysis varies from 0.5 h to 1.9, 16 or 17 h, when ASA is in human blood, human plasma, human gastric juice, or human duodenal juice, respectively [39]. No additional variations were observed in the PL and PLE spectra after 342 min (5.7 h) of exposure of samples to UV light. 

**Figure 2:**
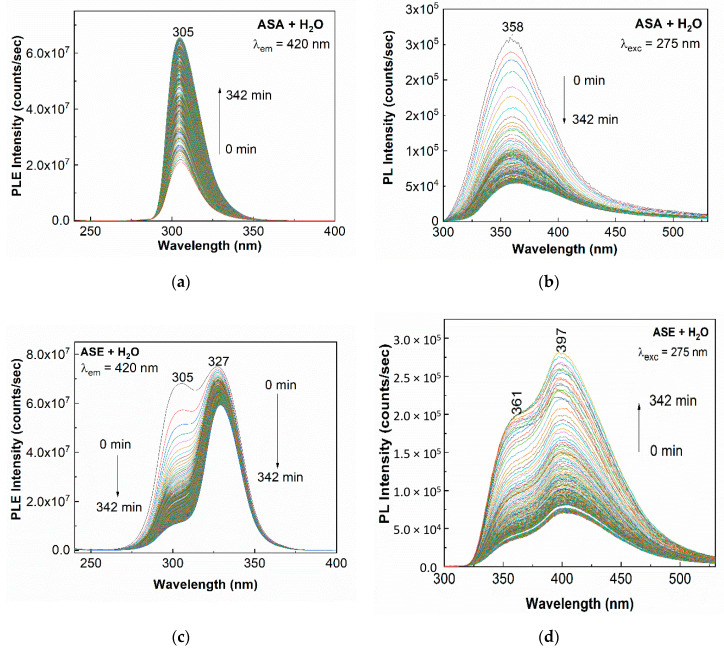
PLE and PL spectra recorded under the emission wavelength of 420 nm and the excitation wavelength of 275 nm, respectively, of the aqueous solution of ASA 0.3 M (**a**,**b**) and ASE 0.3 M (**c**,**d**).

In comparison with the powder state, in the case of the aqueous solutions of ASA and ASE, the following differences were noticed before exposure to UV light of the two samples: (1) The PLE and PL spectra of ASA were shifted at 305 nm and 358 nm, respectively (Figure 2a,b). (2) The PLE spectrum of ASE highlighted two bands with the maxima at 305 nm and 327 nm (Figure 2c). The ratio between the intensities of the two bands at 305 nm ad 327 nm (I_305_/I_327_) became equal to ~0.91. (3) The PL spectrum of ASE showed an intense band with the maximum at 403 nm having a shoulder at 358 nm (Figure 2d). The ratio between the intensities of the two bands at 358 and 403 nm (I_358_/I_403_) was equal to 0.48.

**Figure 3:**
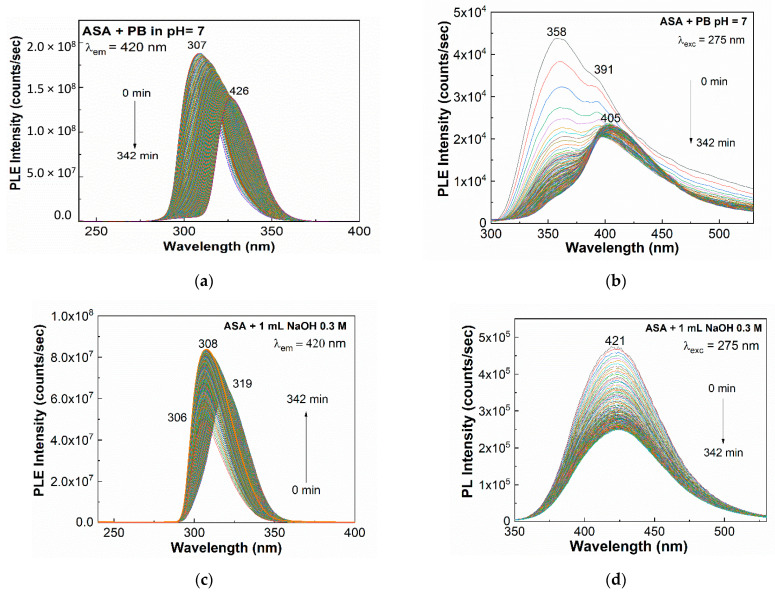
PLE and PL spectra of ASA in the presence of phosphate buffer (PB) with pH = 7 (**a**,**b**, the ASA concentration was 0.3 M) and after the interaction of 2 mL ASA 0.3 M with 1 mL NaOH 0.3 M (**c**,**d**). All PLE and PL spectra were recorded under the emission wavelength equal to 420 nm and the excitation wavelength equal to 275 nm, respectively.

The exposure at the UV light, time of 342 min, induced in: (1) the PLE spectra of ASA, a gradual increase in the intensity of the band, peaked at 305 nm from 2.1 × 10^7^ counts/sec to 6.55 × 10^7^ counts/sec (Figure 2a); (2) the PL spectra of ASA, having maximum at 358 nm, a gradual decrease in the intensity from 3 × 10^5^ counts/sec to 5.5 × 10^4^ counts/sec (Figure 2b); (3) the PLE spectra of ASE, a decrease of the two bands peaked at 305 nm and 327 nm from 6.81 × 10^7^ counts/sec and 7.5 × 10^7^ counts/sec to 1.13 × 10^7^ counts/sec and 5.93 × 10^7^ counts/sec, respectively, so that the ratio between the intensities of the two bands (I_305_/I_327_) varied from 0.91 to 0.19 (Figure 2c); and (4) the PL spectra of ASE, an increase in the intensity of the two emission bands at 358 and 403–400 nm, simultaneously with a variation of the I_358_/I_403_ ratio from 0.48 to 0.69 (Figure 2d).

Under UV light, depending on the medium used for the dissolution of ASA, one observes that in the case of: (1) ASA in PB with pH = 7, an increase in the intensity of the PLE spectra from 1.68 × 10^8^ counts/sec to 1.88 × 10^8^ counts/sec occurred in the first 17 min, followed by a decrease in intensity to ~1.38 × 10^8^ counts/sec, simultaneously with a gradual shift of the maximum of the PLE band from 307 nm to 426 nm (Figure 3a); (2) ASA in PB with pH = 7, the PL spectrum showed two components having maxima at 358 nm and 391 nm, whose intensity gradually decreased from 43.807 counts/sec and 34.644 counts/sec to 6.612 counts/sec and 22.863 counts/sec, respectively, so that the ratio between the intensities of the two bands (I_358_/I_391_) varied from ~1.26 to ~0.29 (Figure 3b); (3) ASA, which interacted with NaOH, caused an increase in the intensity of PLE spectra from 4.81 × 10^7^ counts/sec to 8.34 × 10^7^ counts/sec, succeeded by a decrease in the intensity up to 6.49 × 10^7^ counts/sec; this variation was accompanied by a shift of the PLE band from 306 nm to 319 nm (Figure 3c); and (4) ASA interacted with NaOH, a progressive decrease in the intensity of PL band from 4.73 × 10^5^ counts/sec to 2.5 × 10^7^ counts/sec was reported (Figure 3d).

**Figure 4:**
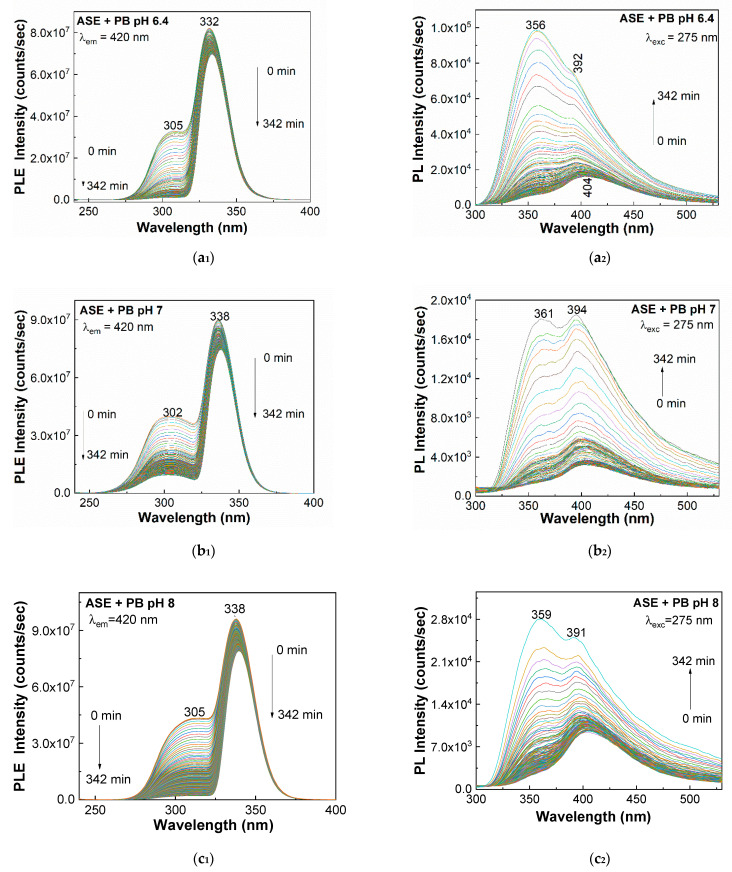
PLE (1) and PL (2) spectra of ASE in the presence of PB with pH equal to 6.4 (**a_1_**,**a_2_**), 7 (**b_1_**,**b_2_**) and 8 (**c_1_**,**c_2_**). PLE and PL spectra were recorded at the emission wavelength equal to 420 nm and the excitation wavelength equal to 275 nm, respectively.

Figure 4 highlights the dependence of the PLE and PL spectra of ASE as function of the PB pH value. 

As increasing the pH from 6.4 to 7 and 8 in Figure 4a_1_,b_1_,c_1_ was highlighted: (1) Prior to exposure of the ASE solution at UV light, all PLE spectra were characterized by an intense band with the maximum at 332–338 nm having the intensity equal to 8.18 × 10^7^ counts/sec, 9 × 10^7^ counts/sec, and 9.61 × 10^7^ counts/sec, accompanied by another band at 305–310 nm whose intensity was equal to 3.26 × 10^7^ counts/sec, 4 × 10^7^ counts/sec, and 4.32 × 10^7^ counts/sec. Thus, before exposure of ASE in PB with pH of 6.4, 7, and 8, the ratio between the intensities of the bands with maxima at 302–305 nm and 332–338 nm (I_302–305_/I_332–338_) was equal to 0.4, 0.44, and 0.45, respectively. (2) As the exposure time of ASE in PB with a pH equal to 6.4, 7, and 8 to UV light increased up to 342 min, in Figure 4a_1_,b_1_,c_1_ a decrease in the intensity of the band at 332–338 nm was observed up to 6.98 × 10^7^ counts/sec, 7.45 × 10^7^ counts/sec, and 8 × 10^7^ counts/sec, while the intensity of PLE band at 305–310 nm was equal to 1.23 × 10^6^ counts/sec, 9.55 × 10^6^ counts/sec, and 2.05 × 10^7^ counts/sec, respectively. After exposure at UV light time of 342 min of ASE in PB with pH of 6.4, 7, and 8, the I_302–305_/I_332–338_ ratio was equal to 0.017, 0.016, and 0.12. According to Figure 4a_2_,b_2_,c_2_, the following variations were seen: (1) Before exposure of ASE in PB with pH equal to 6.4, 7, and 8, to UV light, all PL spectra were characterized by two bands having the maxima at 356–361 nm and 403–404 nm, the ratio between the intensities of the two PL bands being equal to 0.3, 0.4, and 0.3, respectively. (2) After the exposure ASE in PB with pH equal to 6.4, 7, and 8, to UV light, a shift of the emission band at 403–404 nm at 391–394 nm took place. As increasing the exposure time at UV light up to 342 min, the ratio between the intensities of the PL bands at 356–361 nm and 391–394 nm became equal to 1.3, 1, and 1.1, respectively. 

**Figure 5:**
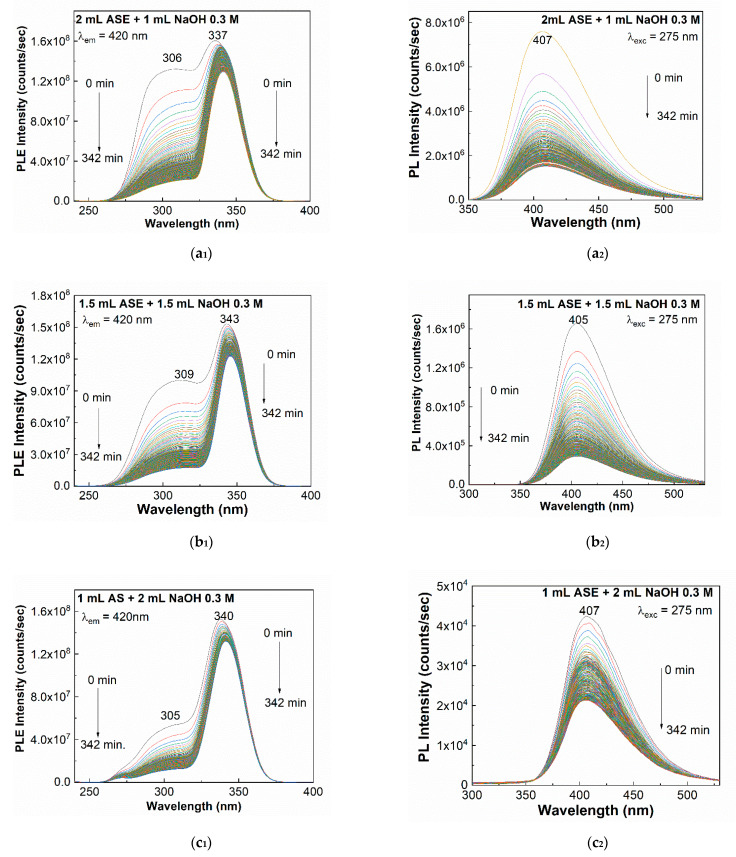
PLE (1) and PL (2) spectra of ASE interacted with NaOH 0.3 M in the volumetric ratio: 2:1 (**a_1_**,**a_2_**), 1.5:1.5 (**b_1_**,**b_2_**), and 1:2 (**c_1_**,**c_2_**). PLE and PL spectra were recorded at the emission wavelength equal to 420 nm and the excitation wavelength equal to 275 nm, respectively.

Similar variations were reported when the aqueous solution of ASE 0.3 M interacted with the solution of NaOH 0.3 M. Figure 5 is relevant in this sense.

**Scheme 1:**
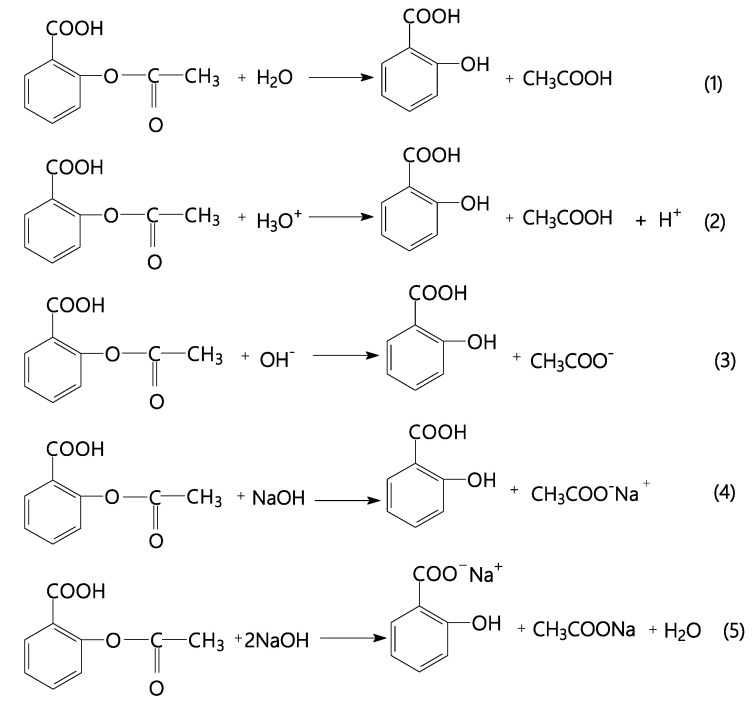
The chemical reactions of ASA with H_2_O and NaOH.

A careful analysis of Figure 5a_1_,b_1_,c_1_, in contrast with Figure 2c, highlighted the following variations: (1) In the initial state, i.e., before exposure at UV light, as weight of NaOH increased in the presence of the same ASE weight, the intensity of the band at 306 nm decreased, so that the ratio between the intensities of the bands at 305–309 nm and 335–343 nm (I_305–309_/I_335–343_) was changed from 0.91 (Figure 2c) to 0.8 (Figure 5a_1_), 0.65 (Figure 5b_1_), and 0.35 (Figure 5c_1_). (2) As increasing the exposure time to UV light, a significant decrease in the intensity of the band at 305–309 nm was seen, the fact which induced a change in the I_305–309_/I_335–343_ ratio from 0.19 (Figure 2c) to 0.14 (Figure 5a_1_), 1.37 (Figure 5b_1_), and 1.5 (Figure 5c_1_). These changes were accompanied in Figure 5a_2_,b_2_,c_2_ by the following variations: (1) Before exposure to UV light, as an increasing in the weight of NaOH added to the ASE, a decrease in the intensity of the PL band at 405–406 nm from 7.6 × 10^6^ counts/sec (Figure 5a_2_) to 1.7 × 10^6^ counts/sec (Figure 5b_2_) and 4.2 × 10^4^ counts/sec (Figure 5c_2_) took place. (2) After 342 min of exposure to UV light, the intensity of the PL spectra in Figure 5 became equal to 1.5 × 10^6^ counts/sec (Figure 5a_2_), 2.9 × 10^6^ counts/sec (Figure 5b_2_), and 2.1 × 10^6^ counts/sec (Figure 5c_2_). The ASA hydrolysis reactions as function of the pH range were studied from the point of view of the activation energy, as from 1952 [40]. We noted that the variation of PLE and PL spectra, above reported, originated in the reactions shown in Scheme 1. Thus, (1) Reaction 1 in Scheme 1 showed chemical process that can be invoked in the case of Figure 2, Figure 3a,b and Figure 4b_1_,b_2_, i.e., when the aqueous solutions of ASA or ASE have pH = 7; (2) Reaction 2 in Scheme 1 illustrated chemical process that induces variations, shown in Figure 4a_1_,a_2_; (3) Reaction 3 in Scheme 1 highlighted the chemical process that led to changes shown in Figure 4c_1_,c_2_; and (4) reactions 4 and 5 in Scheme 1 evidenced the reaction of ASA with NaOH, shown in Figure 3c,d and Figure 5. According to Scheme 1, the reaction products corresponded to salicylic acid (SAL), acetic acid, sodium salicylate, and sodium acetate. 

Taking into account Scheme 1, we think that the different behavior of the PL spectra of ASA and ASE, during the exposure to UV light, originated also in a phototautomerization process of SAL [41], a compound, which in the commercial drugs, there is up to 0.1% [42]. 

**Figure 6:**
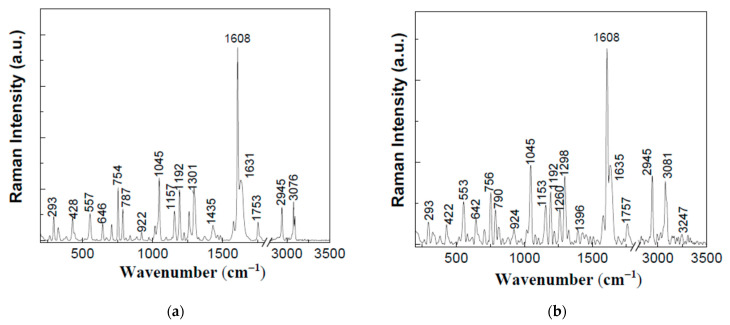
The Raman spectra of ASA in the initial state (**a**) and after the interaction with NaOH (**b**).

**Figure 7:**
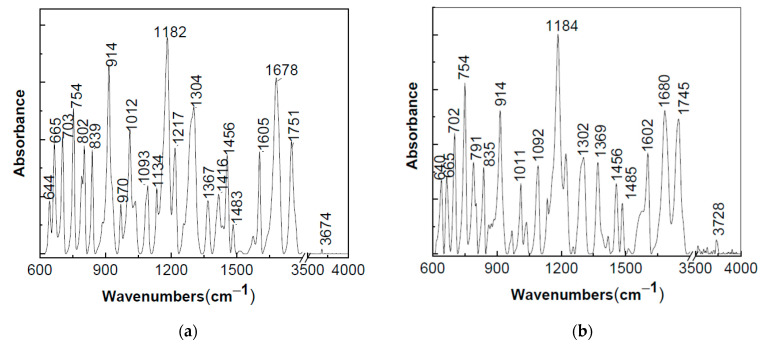
The IR spectra of ASA in the initial state (**a**) and after interaction with NaOH (**b**).

Figure 6 and Figure 7 show the Raman and FTIR spectra of AS in the initial state and after the interaction with NaOH. Figure 6a shows the Raman spectrum of ASA. The main Raman lines of ASA peaked at 557, 646–754, 787, 1045, 1157, 1192, 1260–1301, 1435, 1608, 1631, 1753, 2945, and 3076 cm ^−1^. They were assigned to the following vibrational modes: CO_2_ rocking vibration + bending vibrations for CO_2_, torsion benzene ring, C-H out-of-plane bending, benzene breathing, C-C stretching + C–H in-plane bending in benzene, C–H in-plane bending in benzene, C-C stretching + C–H in-plane bending in benzene, O–H in-plan bending, C-C stretching in benzene ring, C=C stretching in benzene ring, C=O stretching, CH_3_ asymmetrical stretching, and CH_3_ symmetrical stretching, respectively [24,43]. According to Figure 6b, the following changes were observed when ASA interacted with NaOH: (1) a downshift of the Raman lines from 557 cm^−1^ to 553 cm^−1^; (2) a change of the ratio between the intensities of Raman lines peaked at 1608 cm^−1^ and 557 cm^−1^ (I_1608_/I_557_) or 2945 cm^−1^ (I_1608_/I_2945_) from 7.5 and 5.9 to 4.62 and 2.97, respectively; and (3) an upshift of the Raman line from 3076 cm^−1^ to 3081 cm^−1^. These experimental facts indicate change in the molecular structure of ASA when this interacts with NaOH.

Additional arguments concerning the interaction of ASA with NaOH are shown in Figure 7.

The main IR bands of ASA peaked at 703–754–802–839, 914, 970, 1012–1093, 1182, 1217, 1304, 1367, 1415, 1456, 1605, 1678, 1751, and 3674 cm^−1^ (Figure 7a), with these being assigned to the vibrational modes: C–H out-of-plane bending, O-H out-of-plane bending, O-H asymmetrical bending, in-plane bending C-H + stretching C-O in ester, in-plane bending C-H, in-plane bending C-H + stretching C-O, stretching C-O, symmetrical deformation of CH_3_, O-H out-of-plane bending, asymmetrical deformation of CH_3_, stretching C=C in benzene ring, stretching C=O in ester group, and stretching C=O in acid group and OH [24]. After a careful analysis of Figure 7a,b, the following changes were observed: (1) a shift of the IR band from 1751 cm^−1^ to 1745 cm^−1^; (2) a decrease in the absorbance of the IR band peaked at 1678–1680 cm^−1^, which induced a diminution of the ratio between the absorbances of the IR bands peaked at 1678–1680 cm^−1^ and 1751–1745 cm^−1^ from 1.58 to 1.05; and (3) the gradual decrease in the absorbance of the IR bands in the spectral ranges 1000–1100 cm^−1^ assigned to the vibrational mode in-plane bending C-H + stretching C-O in ester as well as of the IR bands peaked at 1304 cm^−1^. These variations indicated the diminution of the weight of the ester groups, as a consequence of the reactions 4 and 5 shown in Scheme 1. 

**Figure 8:**
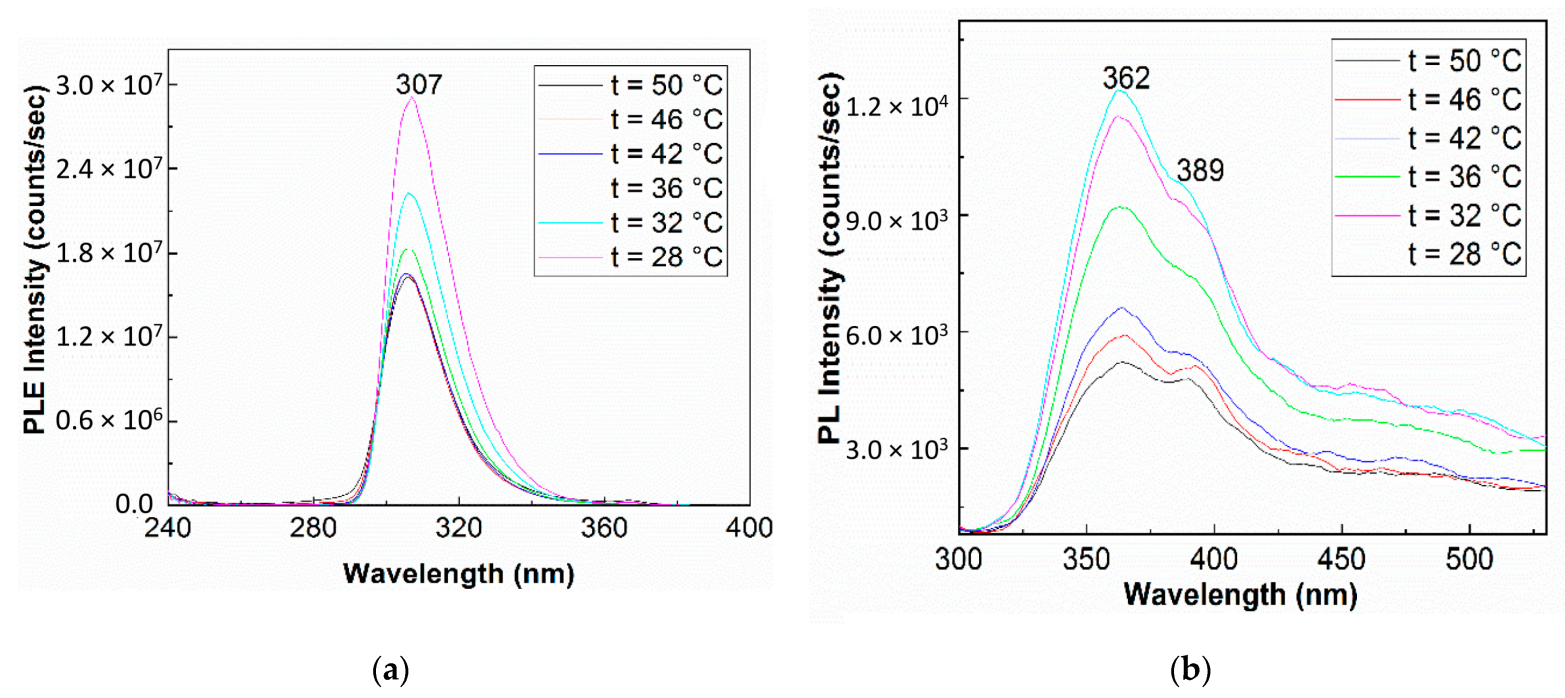
The dependence of the PLE (**a**) and PL (**b**) spectra of the aqueous solution of ASA 0.3 M with the temperature. All PLE spectra were recorded at the emission wavelength of 420 nm, while the PL spectra were recorded at the excitation wavelength of 275 nm.

Figure 8 highlights the dependence of PLE and PL spectra of the aqueous solution of ASA 0.3 M as function of the temperature. 

According to Figure 8a,b, by the heating of the aqueous solution of ASA 0.3 M up to 50 °C one observed that the intensity of the PLE and PL spectra became equal to 1.6 × 10^6^ counts/sec and 5.23 × 10^3^ counts/sec, respectively. These values were smaller in the comparison with those reported immediately after preparing the solution, i.e., 2.1 × 10^7^ counts/sec (Figure 2a) and 3 × 10^5^ counts/sec (Figure 2b). The gradual cooling of the aqueous solution of ASA 0.3 M from 50 °C to 28 °C induced an increase in the intensity of: (1) the PLE spectra from 1.6 × 10^6^ counts/sec to 2.9 × 10^6^ counts/sec (Figure 8a) and (2) the PL spectra of ASA, from 5.2 × 10^3^ counts/sec to 1.22 × 10^4^ counts/sec (Figure 8b). These results demonstrated that the heating–cooling process is not a reversible one, the heating favoring the formation of SAL.

**Figure 9:**
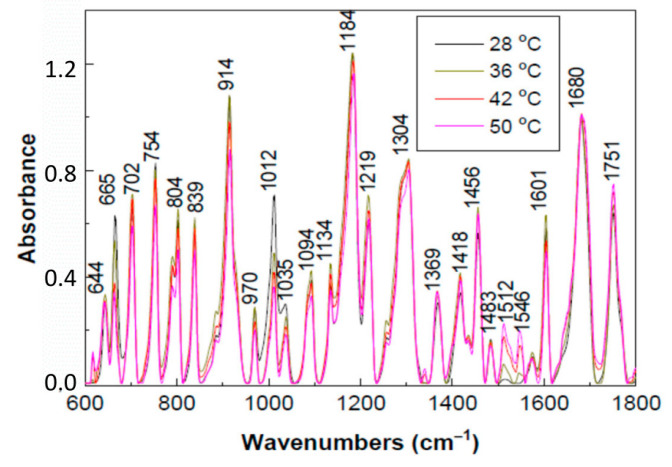
The dependence of the IR spectra of ASA with the temperature.

In order to further argue the optical processes of ASA that take place in the temperature range 28–50 °C, Figure 9 shows the dependence of IR spectra of ASA with temperature. The following changes are observed in Figure 9 as increasing the temperature: (1) a progressive decrease in the absorbance of the IR bands peaked at 1012 cm^−1^ assigned to the vibrational mode in-plane bending C-H + stretching C-O in ester group [24]; (2) change the ratio between the absorbances of the IR bands peaked at 1680 cm^−1^ and 1751 cm^−1^, assigned to the vibrational modes of stretching C=C in benzene ring and stretching C=O in ester group [24], from 1.58 to 1.35; and (3) a gradual decrease in the ratio between the absorbance of the IR bands situated at 914 cm^−1^ and 1680 cm^−1^, attributed to the vibrational modes of O-H out–of-plane bending and stretching C=C in benzene ring [24], from 1.081 to 0.88, simultaneously with the increase of the absorbance of the IR bands peaked at 1512 cm^−1^ and 1546 cm^−1^, assigned to the vibrational mode phenyl-C stretching + deformation of phenyl-H and symmetric deformation of CH_3_ group [44].The first two variations were also reported when ASA was interacted with NaOH, when SAL and CH_3_COOH resulted.

The decrease of the ratio between the absorbance of the IR bands situated at 914 cm^−1^ and 1680 cm^−1^ as well as with the increase of the absorbance of the IR bands peaked at 1512 cm^−1^ and 1546 cm^−1^ indicated a diminution of the OH groups simultaneously with the increase of the weight of the phenyl and CH_3_ groups. These changes can be explained only if we accept the generation of a secondary product of the type of the organic acid anhydride (R-CO-O-CO-R, where R corresponds to C_6_H_4_-O-CO-CH_3_) took place. These preliminary results on ASA changes with temperature require, in the next stage, additional clinical trials to consider, in addition, the electrolyte imbalance in the case of patients with various gastrointestinal, cardiovascular, and lung diseases [45].

## 3. Materials and Methods

Acetylsalicylic acid (ASA), NaOH, Na_2_HPO_4_, and NaH_2_PO_4_ were purchased from Sigma Aldrich. Sodium hydroxide (NaOH), 98% purity, was purchased from Alfa Aesar. The tablets of aspirin (ASE), having as (1) active compound 500 mg ASA and (2) excipients, corn starch and cellulose powder, were bought from local pharmacies. 

The PB solutions with pH equal to 6.4, 7, and 8 were prepared using the aqueous solutions of Na_2_HPO_4_ and NaH_2_PO_4_. In order to highlight the photodegradation of ASA and ASE, we prepared (1) an aqueous solution of ASA 0.3 M and (2) another of AS with the concentration of ~0.3 M by dissolving of a tablet of ASE, having as active compound 500 mg ASA in 10 mL distilled water or PB, under ultrasonication, for 10 min. In order to remove water-insoluble excipients, a successive filtration was carried out. 

The photodegradation of ASA and ASE was highlighted in this work by hydrolysis reaction of ASA or ASE with alkaline media such as the aqueous solutions of NaOH 0.3 M.

The photodegradation of ASA and ASE under UV light, for 362 min, was studied by photoluminescence, IR spectroscopy, and Raman scattering. In the case of the last two analyses, the photodegradation studies were carried out with a mercury-vapor lamp having the power of 350 W, purchased from Newport company (Irvine, CA, USA). The irradiation power was of 4.46 mW/cm^2^.

The photoluminescence (PL) and photoluminescence excitation (PLE) spectra of the ASA and ASE solutions were recorded in right-angle geometry, at room temperature, using a Fluorolog -3 spectrometer, model FL3–22, from Horiba Jobin Yvon, which was endowed with a Xe lamp with the power of 450 W, purchased from Newport company (Irvine, CA, USA). The irradiation power was 4.42 mW/cm^2^. 

The devices used for the exposure to UV light of the samples were equipped with a cooling system. Raman spectra of ASA in the initial state and after the interaction with NaOH were recorded, at the excitation wavelength of 1064 nm, with a FT Raman spectrophotometer, RFS100S model, from Bruker ((Bruker Optik GmbH, Ettlingen, Germany)).

IR spectra of ASA in the initial state and after the interaction with NaOH were recorded with a FTIR spectrophotometer, Vertex 70 model, from Bruker (Billerica, MA, USA).

## 4. Conclusions

In this work, new results are reported concerning the photodegradation of ASA and ASE, by PL, Raman scattering, and FTIR spectroscopy. Our results allow us to conclude that (1) PL is a valuable tool to highlight the presence of SAL in commercial drugs; (2) the monitoring of the hydrolysis reaction of the solutions of ASA 0.3 M in PB with pH equal to 6.4, 7, and 8 induced a gradual increase in the intensity of PLE spectra simultaneously with a progressive decrease of the intensity of PL spectra; (3) the hydrolysis reaction of the solutions of ASE 0.3 M in PB with pH equal to 6.4, 7, and 8 showed a different behavior, as a consequence of the presence of SAL, when a phototautomerization process took place, too; (4) the interaction of ASA with NaOH led to a decrease of the ester groups in the favor of carboxylic groups, as shown by Raman scattering and FTIR spectroscopy; and (5) considering the smaller intensities of the PLE and PL spectra of the aqueous solution of ASA after the heating–cooling process, an irreversible character was invoked in this case.

## Data Availability

Samples of ASA and ASE are available from the authors.
